# Supplementary material for: Identification of the SARS-unique domain of SARS-CoV-2 as an antiviral target
Source: Nat Commun. 2023 Jul 6;14:3999. doi: 10.1038/s41467-023-39709-6 (PMC10326071; doi:10.1038/s41467-023-39709-6)
Supplement: Supplementary file 1 — Supplementary information [file 41467_2023_39709_MOESM1_ESM.pdf]

Supplementary materials of

Identification of the SARS-Unique Domain of SARS-CoV-2 as an Antiviral Target

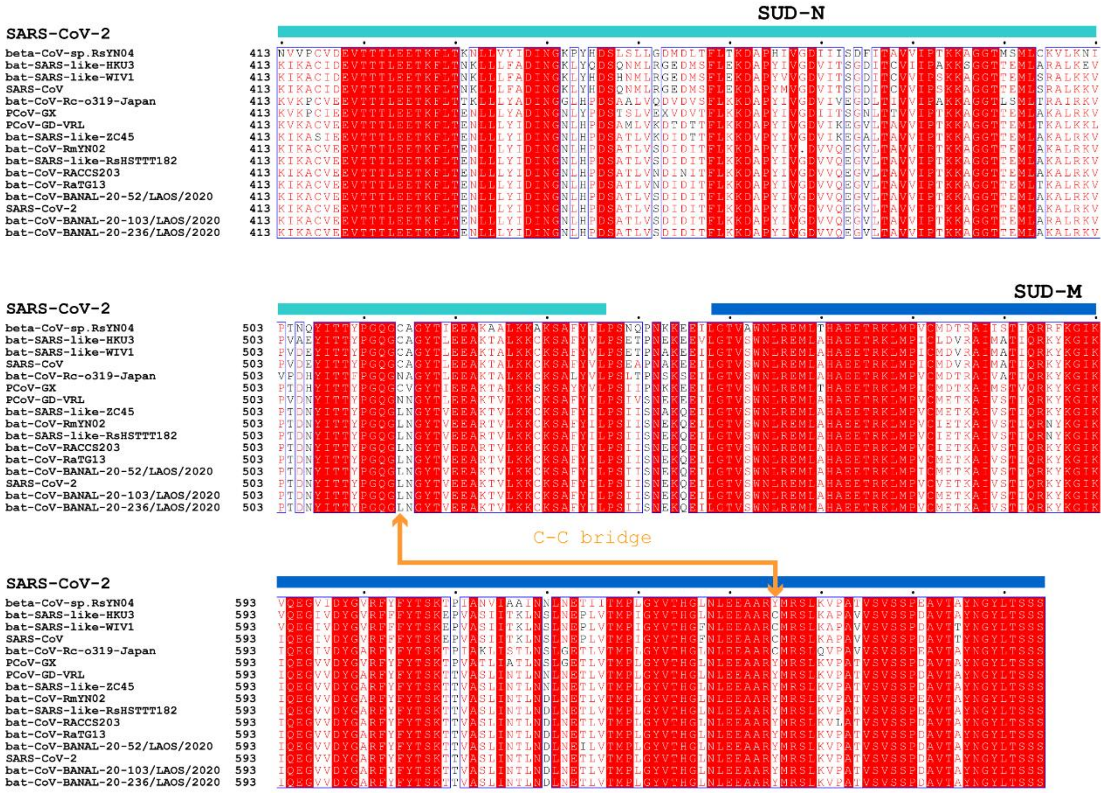

Supplementary Figure 1. Multiple sequence alignment of SUD-core from a selection of Sarbecoviruses

Multiple sequence alignment of a selection of Sarbecovirus SUD-core (aligned by Clustal omega and rendered by ESPript). SUD-N and SUD-M domains are indicated by cyan bar and blue bar. The disulfide bond formed between SARS-CoV SUD-N and SUD-M is indicated by orange arrow.

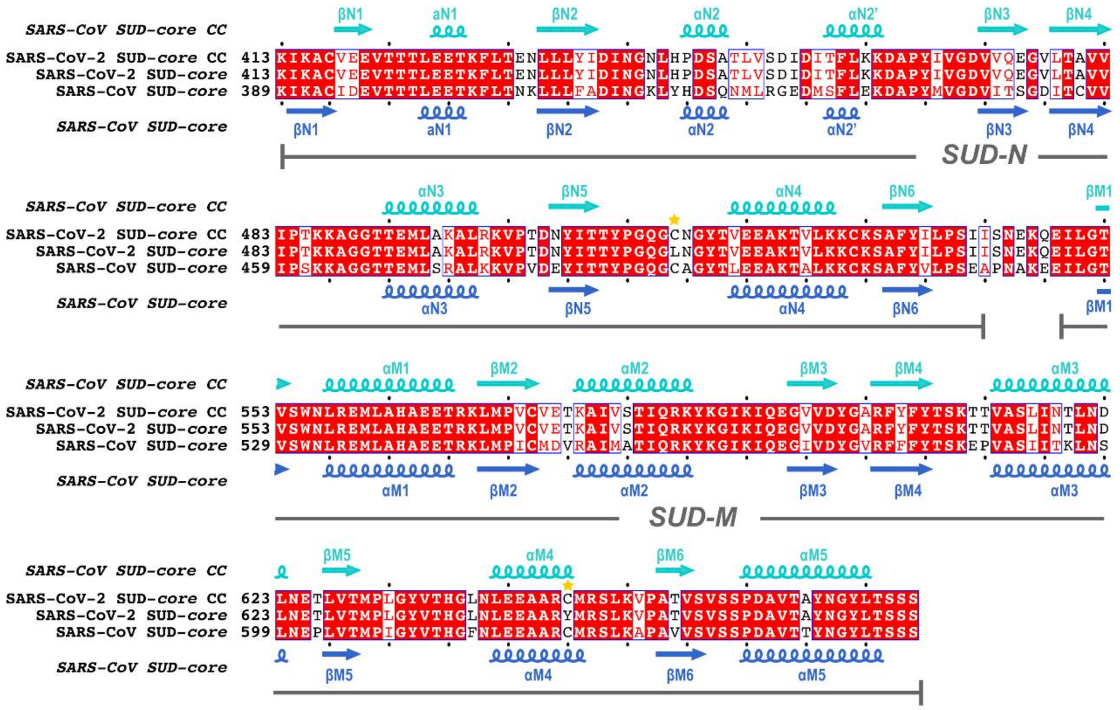

Supplementary Figure 2. Structure-based sequence alignment of SARS-CoV SUD-core, SARS-CoV-2 SUD-core and SARS-CoV-2 SUD-core-CC

Amino acid sequences of SUD-core from SARS-CoV and SARS-CoV-2 were aligned and the secondary structural elements of SARS-CoV-2 SUD-core-CC (this study, cyan) SARS-CoV SUD-core (PDB: 2W2G, blue) are superimposed on top and bottom of the sequences. SUD-N and SUD-M domain are indicated. Disulfide forming residues are indicated by orange stars.

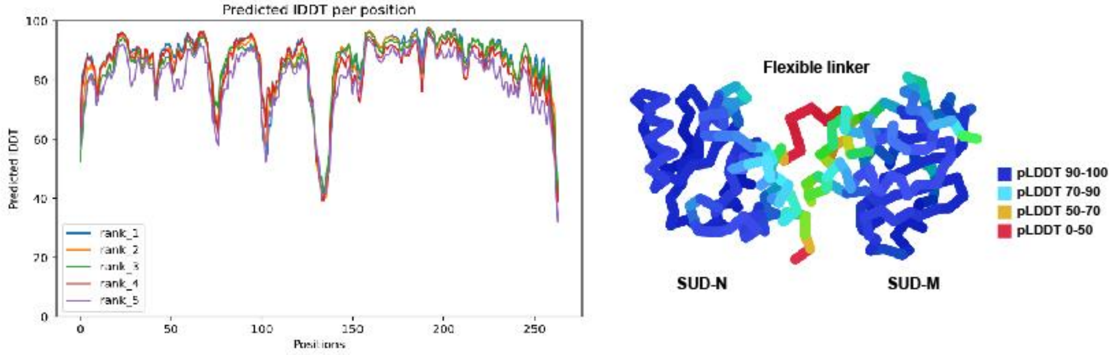

Supplementary Figure 3. Prediction of SARS-CoV-2 SUD-core using AlphaFold 2

Left, pLDDT scores are plotted as the function of each residue of five highly ranked Alphafold2 model (rank 1-5). Right, ribbon representation of the highest ranked model (rank 1), colored by pLDDT score. The linker region connecting SUD-N and SUD-M has the lowest pLDDT, implying the structure prediction here is unreliable.

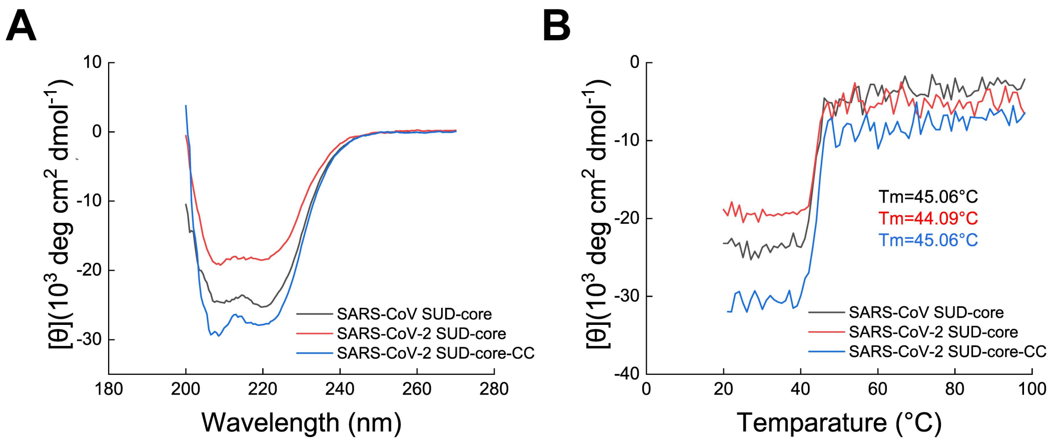

Supplementary Figure 4. Circular dichroism (CD) spectroscopy of various SUD proteins

A. CD spectra of SARS-CoV SUD-core (black), SARS-CoV-2 SUD-core (red) and SARS-CoV SUD-core-CC (blue) were recorded under oxidative conditions.

B. Thermostability measurement of the indicated SUD-core proteins using CD spectroscopy. SARS-CoV SUD-core (black) exhibits lower melting temperature (Tm) than that of SARS-CoV SUD-core-CC (blue) or SARS-CoV-2 SUD-core (red), which contains a disulfate bridge between SUD-N and SUD-M.

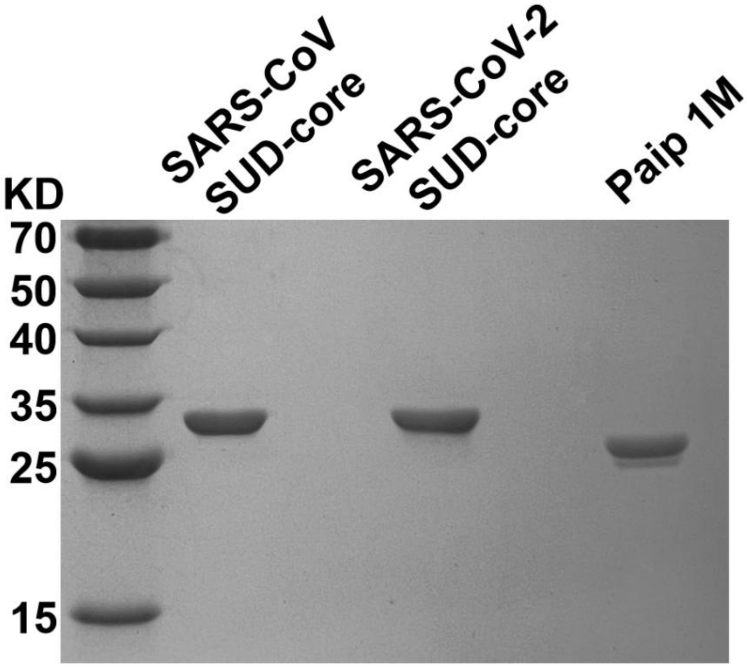

Supplementary Figure 5. SDS-PAGE analyses finally purified SARS-CoV SUD-core, SARS-CoV-2 SUD-core and Paip1M

SARS-CoV-2 SUD-core, SARS-CoV SUD-core and Paip1M were overexpressed in E. coli cells and purified. The Final products were analyzed by SDS-PAGE stained with Coomassie blue. Molecular standards are indicated. Each experiment was repeated three times independently with similar results.

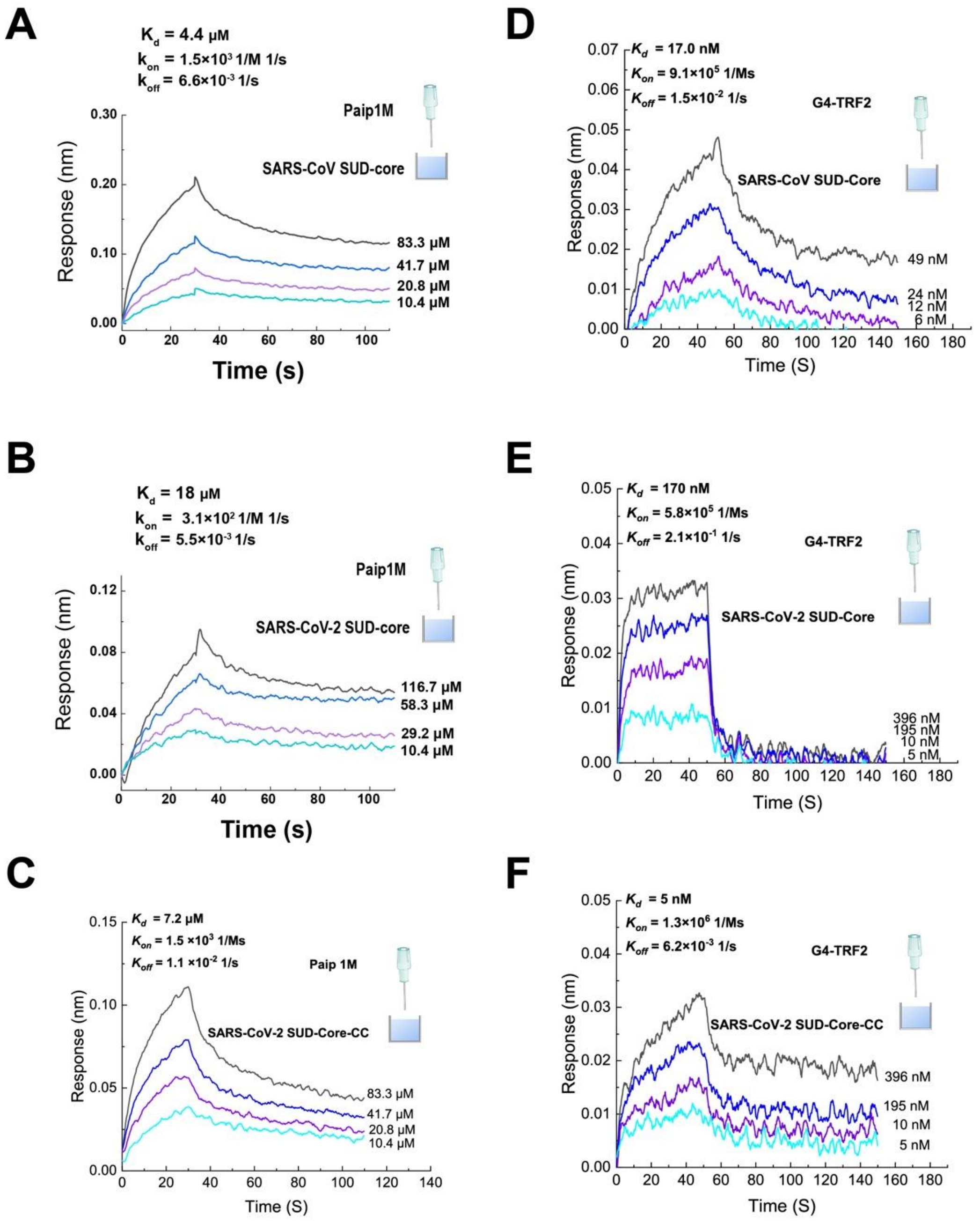

Supplementary Figure 6. SUD-Paip 1M binding and SUD-G4 RNA binding investigated using BLI titrations.

The interactions between various SUD proteins and Paip 1M were investigated using BLI (A-C). The interactions between various SUD proteins and G4-RNA TRF2 were investigated using BLI (E-F). Sensorgrams, binding kinetic parameters Kd, kon and koff of the BLI titrations are illustrated.

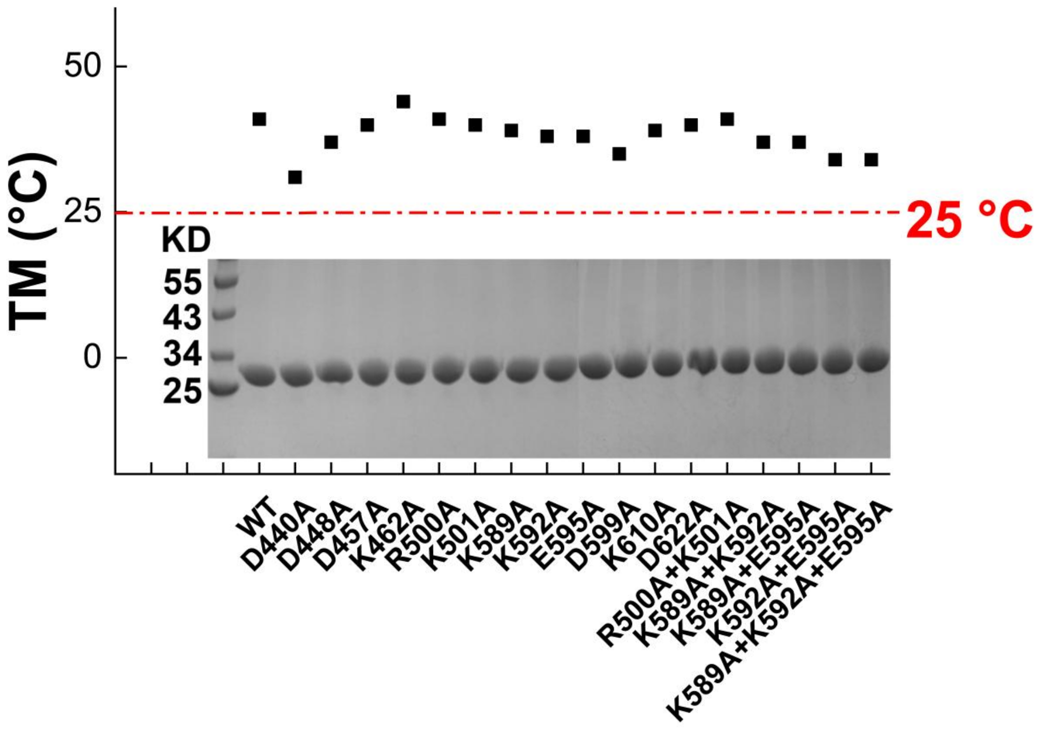

Supplementary Figure 7. Stability assessment of SARS-CoV-2 SUD-core mutants

Thermostability of SARS-CoV-2 SUD-core mutants were measured by thermal shift assay; T_m values are plotted (filled squares). An SDS-PAGE analysis of all mutant proteins is shown below the plots and indicated. All mutants are relatively stable under room temperature, Indicated with a red dashed line. Each experiment was repeated three times independently with similar results.

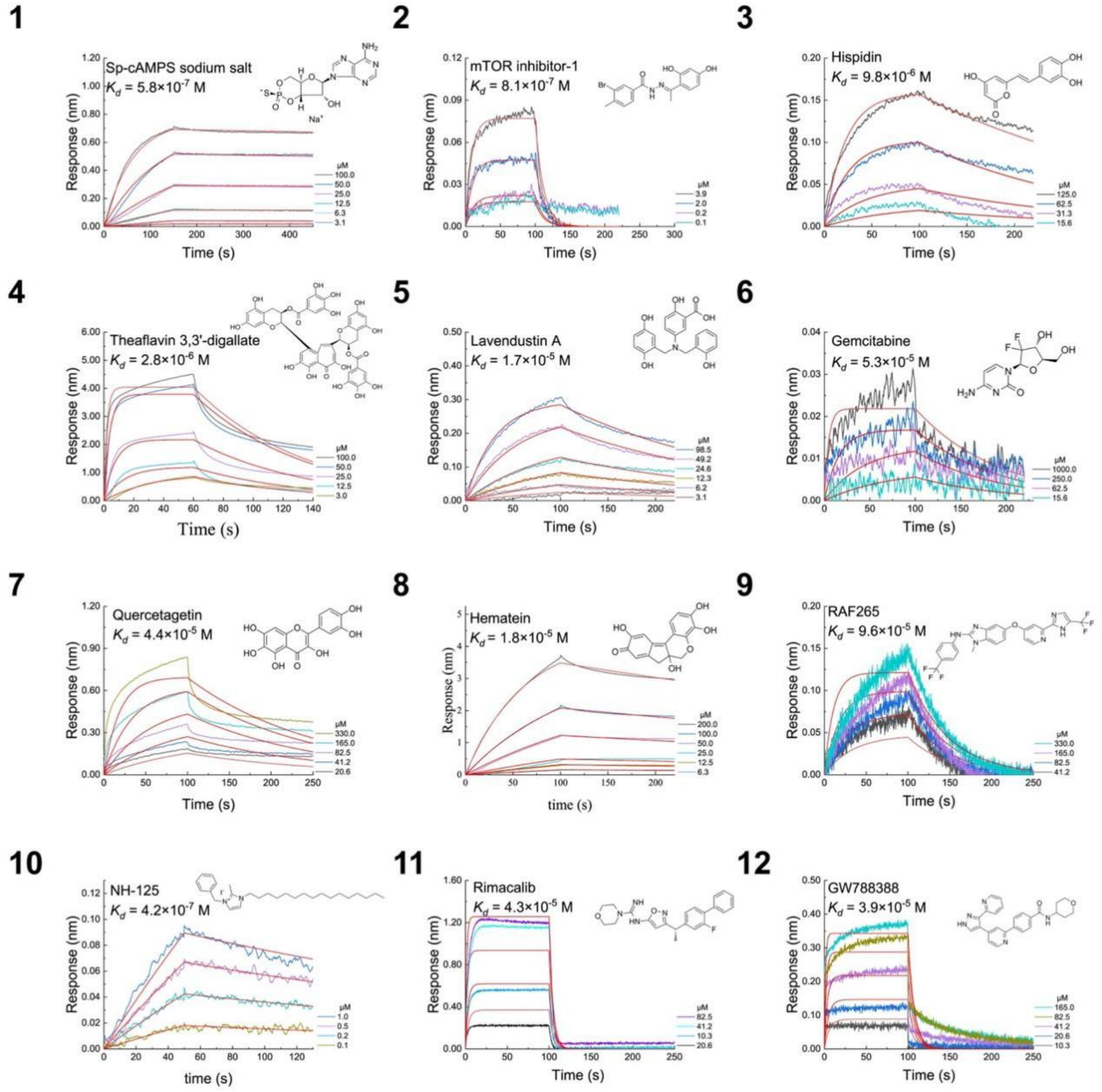

Supplementary Figure 8. Binding kinetics of top 12 hit compounds with the target protein SARS-CoV-2 SUD-core

SARS-CoV-2 SUD-core was biotinylated for immobilization on the SSA biosensors. Top 12 hit compounds (numbered 1-12) were diluted to concentration series in PBS (pH=7.4) for association with immobilized SARS-CoV-2 SUD-core on biosensors for 150s. The following disassociation was 180s in fresh PBS containing 1% DMSO. Data were recorded and processed with the double reference subtraction and local fitting with 1:1 model using ForteBio Data analysis v11.1. The name, chemical structure and disassociation constant K_d of each hit compound are indicated. Finally, Comp.**1-4** were selected for antiviral activity assessment.

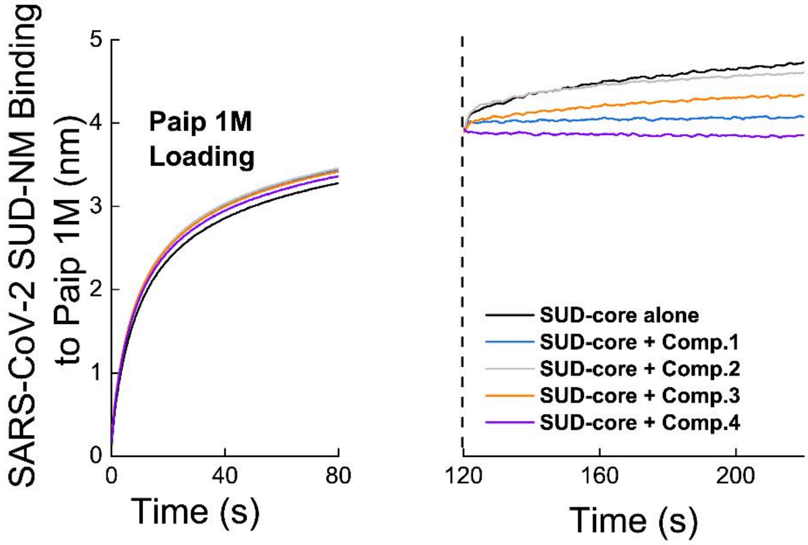

Supplementary Figure 9. Hit compounds undermine SUD-Paip 1M interaction

BLI experiments demonstrating the SUD-Paip 1M interaction was disrupted by four hit compounds to different extents. Biotinylated Paip 1M was loaded to SA biosensors for measuring the binding with SARS-CoV-2 SUD-core in the presence of the indicated hit compounds. BLI responses during association experiments were aligned.

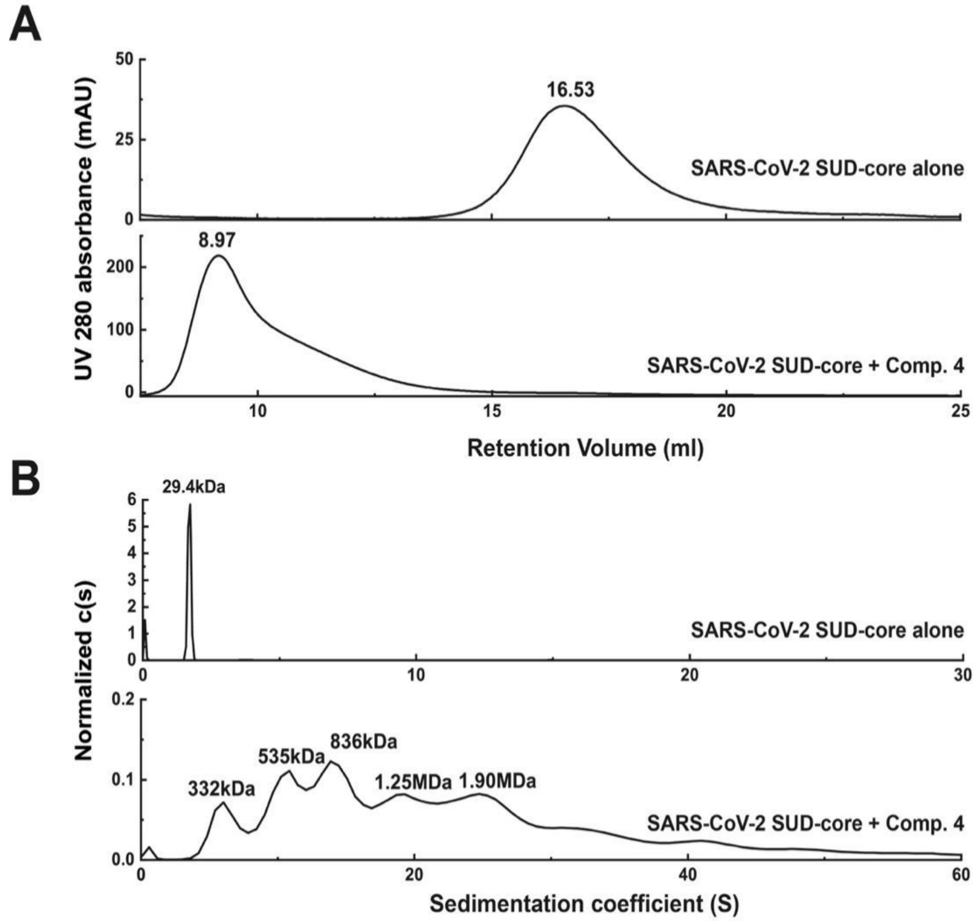

Supplementary Figure 10. Comp.4 disrupts monodispersity of SARS-CoV-2 SUD-core in solution.

A. Size-exclusion chromatographic profiles of SARS-CoV-2 SUD-core alone and complexed by Comp.**4**. SARS-CoV-2 SUD-core eluted as monodispersed species in the absence of Comp.**4**. In the presence of Comp.**4**, the protein eluted as polydispersed species.

B. To reveal details for structural heterogenicity of SARS-CoV-2 SUD-core in the absence and presence of Comp.**4**, we examined the above samples by analytical ultracentrifugation. Consistent with size-exclusion experiments, SARS-CoV-2 SUD-core formed homogenous monomers, whereas Comp. **4** induced SUD-core heterogenous aggregations with large molecular masses. Molecular mass of each peak is indicated.

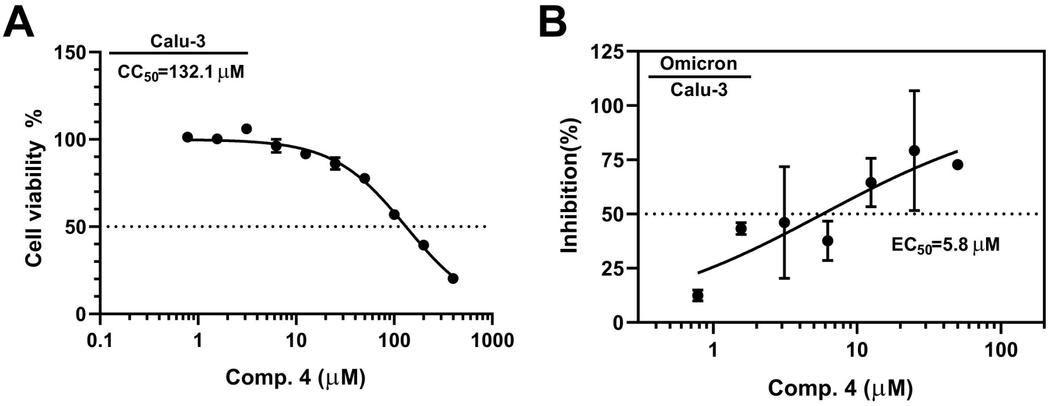

Supplementary Figure 11. Antiviral activity of Comp.4 against SARS-CoV-2 using Calu-3 cells

A. The cytotoxicity of Comp.**4** on Calu-3 cells was measured by the MTT method using the indicated concentrations. All data were shown as mean±SEM, n=3 independent experiment.

B. EC_{50} of Comp.**4** on Calu-3 cells was plotted by a viral load reduction assay. The experiments (n=3) were repeated twice with similar results. All data were shown as mean±SEM,

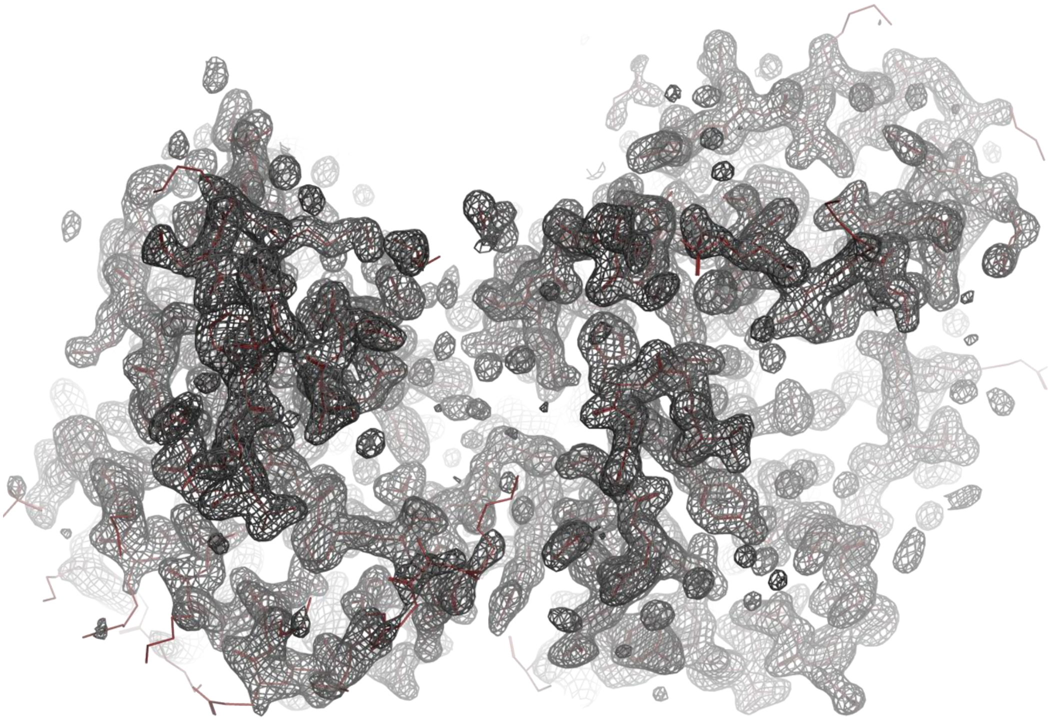

Supplementary Figure 12. Final 2Fo-Fc electron density map of SARS-CoV-2 SUD-core-CC crystal structure (1.58 Å resolution) with superimposed model.

The final 2Fo-Fc electron density map of SARS-CoV-2 SUD-core-CC crystal structure (black mesh, PBD: 8HBL, contour 1.3 σ) is superimposed with the final atomic model (red).

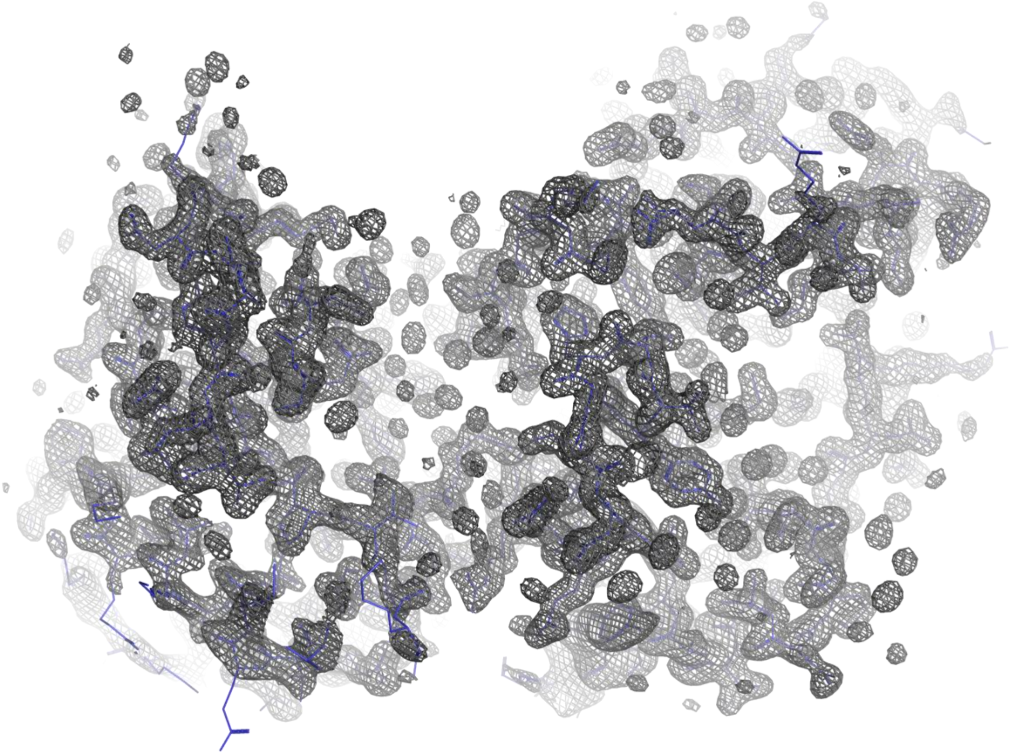

Supplementary Figure 13. Final 2Fo-Fc electron density map of the Comp.4 soaked SARS-CoV-2 SUD-core-CC crystal structure (1.35 Å resolution) with superimposed model.

The final 2Fo-Fc electron density map of the Comp.4 soaked SARS-CoV-2 SUD-core-CC crystal structure (black mesh, PBD: 8GQC, contour 1.3 σ) is superimposed with the final atomic model (blue). Electron densities of Comp.4 was not found in the map.

Supplementary Table 1. Structural comparison among SARS-CoV-2 SUD-core-CC crystal structure, SARS-CoV SUD-core and the Alphafold2 models

RMSD		SARS-CoV-2 SUD-core-CC crystal structure (8GQC)		
		**Core**	**N**	**M**
**SARS-CoV SUD-core (2W2G)**	**Core**	3.0 Å	-	-
	**N**	-	1.7 Å	-
	**M**	-	-	0.8 Å
**SARS-CoV SUD-core AF2 model**	**Core**	5.2 Å	-	-
	**N**	-	1.0 Å	-
	**M**	-	-	0.9 Å
