# Supplementary material for: Identification of the SARS-unique domain of SARS-CoV-2 as an antiviral target
Source: Nat Commun. 2023 Jul 6;14:3999. doi: 10.1038/s41467-023-39709-6 (PMC10326071; doi:10.1038/s41467-023-39709-6)
Supplement: Supplementary file 3 — Description of Additional Supplementary files [file 41467_2023_39709_MOESM3_ESM.pdf]

Description of Additional Supplementary files

File name: Supplementary Data 1
Description: Binding kinetics and thermostability of top-ranked compounds withSARS-CoV-2 SUD-core
