# Supplementary material for: Identification of the SARS-unique domain of SARS-CoV-2 as an antiviral target
Source: Nat Commun. 2023 Jul 6;14:3999. doi: 10.1038/s41467-023-39709-6 (PMC10326071; doi:10.1038/s41467-023-39709-6)
Supplement: Supplementary file 4 — Supplementary Data 1 [file 41467_2023_39709_MOESM4_ESM.pdf]

Supplementary Data 1:

Binding kinetics and thermostability of top-ranked compounds with SARS-CoV-2 SUD-core

Comp. No.	Comp. name	Chemical Structure	Binding kinetics and thermostability with SARS-CoV-2 SUD-core			
			K_d (M)	k_{on} (1/Ms)	k_{off} (1/s)	T_m (°C)
1*	Sp-cAMPS sodium salt	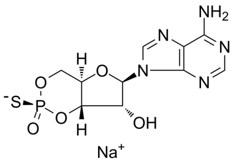	5.8×10^{-7}	1.7×10^{2}	9.6×10^{-5}	36.0
2*	mTOR inhibitor-1	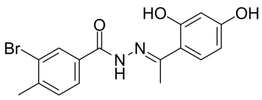	8.1×10^{-7}	8.1×10^{3}	8.5×10^{-2}	40.0
3*	Hispidin	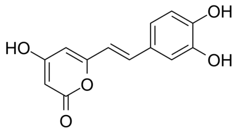	9.8×10^{-6}	5.6×10^{2}	5.5×10^{-4}	39.0
4*	Theaflavin 3,3'-digallate	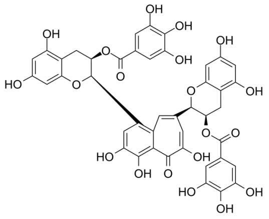	2.8×10^{-6}	4.9×10^{3}	1.3×10^{-2}	37.0
5	Lavendustin A	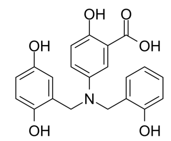	1.7×10^{-5}	2.9×10^{2}	4.9×10^{-3}	38.0
6	Gemcitabine	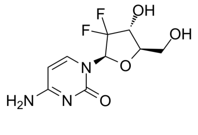	5.3×10^{-5}	2.1×10^{2}	1.1×10^{-2}	36.0

7	Quercetagetin	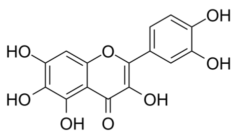	4.4×10^{-5}	1.4×10^{2}	7.5×10^{-3}	36.5
8	Hematein	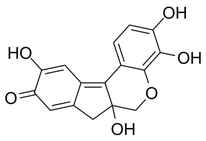	1.8×10^{-5}	7.6×10^{1}	1.4×10^{-3}	35.0
9	RAF265	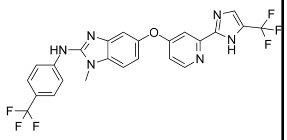	9.6×10^{-5}	5.4×10^{2}	2.3×10^{-2}	39.0
10	NH125	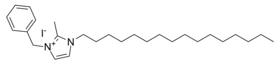	4.2×10^{-7}	9.3×10^{3}	3.9×10^{-3}	32.0
11	Rimacalib	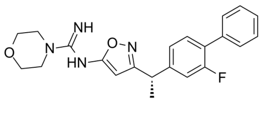	4.3×10^{-5}	1.4×10^{4}	2.6×10^{-1}	37.0
12	GW788388	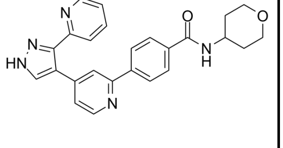	3.9×10^{-5}	5.4×10^{2}	2.2×10^{-2}	36.0

*** hits compounds**
